# Association of red blood cell distribution width combined with alanine aminotransferase with gastrointestinal hemorrhage in patients with acute pancreatitis: a retrospective MIMIC-IV study

**DOI:** 10.3389/fmed.2026.1777755

**Published:** 2026-05-28

**Authors:** Yazhen Zhan, Yaling Wei, Yuke Zhan, Ye Lu, Jinnan Ding

**Affiliations:** 1 Department of Gastroenterology, Shaoxing Central Hospital, Shaoxing, Zhejiang, China; 2 Hangzhou Medical College, Hangzhou, Zhejiang, China

**Keywords:** acute pancreatitis, alanine aminotransferase, gastrointestinal hemorrhage, readmission, red blood cell distribution width

## Abstract

**Background:**

Readmission is common in acute pancreatitis (AP), with a rate of about 20%. Acute kidney injury (AKI), shock, and gastrointestinal (GI) hemorrhage are serious complications related to adverse outcomes in AP. This study aimed to identify the complication most strongly associated with readmission in AP for further risk factor analysis.

**Methods:**

Data were downloaded from the MIMIC-IV database. Chi-square test and the Phi coefficient were used to explore which complication was most strongly associated with readmission in AP. Then, patients were divided into with/without GI hemorrhage groups to observe the baseline characteristics across the two groups. LASSO regression and exploratory machine-learning algorithms involving XGBoost, random forest, and K-nearest neighbor were employed for feature selection. Moreover, restricted cubic spline (RCS) and logistic regression analyses were used to evaluate the associations of the variables with GI hemorrhage.

**Results:**

There were 2,004 participants with AP enrolled with 445 readmission records. Among the three complications examined, GI hemorrhage showed the strongest association with readmission. Twelve parameters related to GI hemorrhage were enrolled for feature selection, with three consistently prioritized variables: age, alanine aminotransferase (ALT), and red blood cell distribution width (RDW). GI hemorrhage had a negative relationship with ALT but was positively linked to RDW (all *P* < 0.05). When combining RDW and ALT, the multivariable regression results showed that AP patients in the low-ALT + high-RDW group exhibited the highest odds ratio (OR) of GI hemorrhage (OR = 2.315).

**Conclusion:**

This study highlights that GI hemorrhage showed the strongest association with readmission among the complications examined in AP. Moreover, RDW and ALT were independently associated with GI hemorrhage risk. The low-ALT + high-RDW pattern may help identify patients at higher risk, but these findings are exploratory and require external validation before clinical application.

## 1 Introduction

Acute pancreatitis (AP) is a prevalent severe digestive disease related to an estimated $2.6 billion in medical costs annually (1). The incidence of AP ranges from 10 to 50 per hundred thousand populations each year (2). The emergency department visits for AP have increased by 18% and over 300,000 annual hospital admissions have been found in AP patients in recent decades (3, 4). There was an increase in hospitalized admissions because of AP from 9.48/1,000 cases in 2002 to 12.19/1,000 cases in 2013, with approximately a median $7,000 hospitalization cost (5, 6). Hospital readmission is a valuable indicator reflecting the quality of patient care. Besides, it imposes a considerable burden on the individual patient as well as the medical healthcare system (7). Hospitals are incentivized to reduce unplanned readmissions for Medicare patients throughout the US. Munigala et al. conducted a study based on the National Readmission Database and reported that 25.7% of discharged AP patients would be readmitted to the hospital (8).

AP is a non-age-specific inflammatory pancreatic disease with gallstones, hypertriglyceridemia, and alcohol abuse as the major causes (9). Premature activation of trypsinogen to trypsin initiates an activation cascade, resulting in pancreatic damage and an inflammatory process (10). Imaging techniques including magnetic resonance imaging and computed tomography, laboratory tests, and clinical presentations are the available methods for AP diagnosis (11). Abdominal pain is the most common symptom of AP with a proportion of nearly 90%; patients may also present with vomiting, distension, breathlessness, and rebound tenderness (12). Most of them had a mild symptom, while 20% of AP cases will progress to severe AP and develop complications, leading to multiple organ failure such as acute kidney injury (AKI), shock, and gastrointestinal (GI) hemorrhage with prolonged disease course and poor prognosis (13). Current approaches for AP treatment are limited without specific targeted drugs (14). Therefore, it is necessary to promptly identify the complications related to readmission and take corresponding measures to improve the life quality of patients and alleviate the social healthcare burden.

Herein, we conducted a retrospective study using the MIMIC-IV database by two-step framework: first, we explored the associations of three major complications (AKI, shock, and GI hemorrhage) with readmission among AP patients, and identified that GI hemorrhage showed the strongest association with readmission in this cohort; second, we analyzed admission factors associated with GI hemorrhage through univariable analysis, Least Absolute Shrinkage and Selection Operator (LASSO) regression, exploratory machine-learning algorithms, restricted cubic spline (RCS), and logistic regression models.

## 2 Materials and methods

### 2.1 Data source and study population

This retrospective study was conducted based on the MIMIC-IV, an updated version of MIMIC-III with pre-existing institutional review board approval. This is a large, comprehensive, and publicly accessible database containing high-quality data of patients admitted to Beth Israel Deaconess Medical Center between 2008 and 2019. Due to the de-identification of patient information, informed consent was not required.

Inclusion criteria: the diagnosis of AP according to the International Classification of Diseases codes versions 9 and 10 (ICD-9; ICD-10), including ICD-9 code 577.0 and ICD-10 code K85; have an age no less than 18 years. Exclusion criteria: with end-stage renal disease (*n* = 49); with cirrhosis (*n* = 78); with malignancy (*n* = 360); length of hospital stay < 24 h (*n* = 103). The selection criteria finally resulted in 2,004 samples for the following analyses.

### 2.2 Variable collection

Data were retrieved by using the structured query language from the MIMIC-IV database. Readmission data was recorded (readmission was defined as a subsequent admission after the discharge of the first admission). Because MIMIC-IV is a single-center database, only readmissions captured within the same medical center could be assessed. The other factors covering complications, demographics, comorbidities, treatment, and admission laboratory indexes such as liver and kidney function, blood tests, and electrolytes were also obtained. In detail, complications consisted of AKI, GI hemorrhage, and shock identified from structured diagnosis records during the index hospitalization; demographics: age (years), gender (male/female), body mass index [BMI, calculated by weight (kg)/height^2^ (m^2^)], race (White/non-White), alcohol use (no/yes), and cigarette use (no/yes); comorbidities identified by ICD codes: diabetes (no/yes), hypertension (no/yes), chronic pulmonary disease (COPD), and mild liver disease; treatment included ventilation use and vasopressin use; admission laboratory indexes were defined as the first available measurements within the 24 h after admission, including alanine transaminase (ALT, U/L), aspartate aminotransferase (AST, U/L), bicarbonate (mEq/L), blood urea nitrogen (BUN, mg/dL), calcium (mg/dL), chloride (mEq/L), creatinine (mg/dL), glucose (mg/dL), platelets (K/uL), potassium (mEq/L), red blood cell distribution width (RDW, %), sodium (mEq/L), total bilirubin (mg/dL), and white blood cell (WBC, K/uL).

### 2.3 Statistical analysis

All statistical analyses were performed using R version 4.4.1 and *P* value < 0.05 was considered statistically significant.

#### 2.3.1 Identification of the most relevant complication

To identify which complication was most strongly associated with readmission, we first performed Chi-square tests between readmission (yes/no) and each complication (AKI, shock, and GI hemorrhage). The strength of association was quantified using Phi coefficient (ϕ) for 2 × 2 contingency tables. The complication with the largest absolute ϕ value and a significant Chi-square test (*P* < 0.05) was considered the most relevant.

#### 2.3.2 Risk factor analysis for GI hemorrhage

After determining GI hemorrhage as the complication most associated with readmission, we performed a risk factor analysis using GI hemorrhage as the binary outcome.

*Baseline comparison between GI hemorrhage groups:* first, patients were divided into two groups according to the presence of GI hemorrhage and baseline comparisons were conducted. The Chi-square test was used for group differences in qualitative data represented by counts (percentages). The Shapiro-Wilk method was adopted for the normality test for measurement data. Those with normal distribution were presented as mean ± standard deviation, otherwise by median [interquartile range]. Group differences were evaluated by the Student's *t*-test and Mann-Whitney-U test accordingly.

*LASSO regression for variable selection:* second, all candidate variables that reached statistical significance in the baseline comparison were entered into a LASSO logistic regression with GI hemorrhage as the outcome. Ten-fold cross-validation was used to select the optimal tuning parameter λ. Variables with non-zero coefficients at the optimal λ were retained as the most relevant predictors of GI hemorrhage.

*Machine learning-based variable importance ranking (exploratory analysis):* third, to explore the relative importance of the LASSO-selected variables, we performed three machine learning algorithms: XGBoost, random forest, and K-nearest neighbor (KNN). The top three features, ALT, age, and RDW, were consistently prioritized across these exploratory algorithms.

*Dose-response analysis:* RCS analysis with three knots was used to describe the association between continuous variables (ALT and RDW) and outcome (GI hemorrhage).

*Multivariable logistic regression models and model validation:* a multivariable logistic regression model was constructed using seven LASSO-selected predictors to examine the independent associations of ALT and RDW with GI hemorrhage. To address concerns about potentially omitted confounders, a sensitivity analysis was performed. In addition to the seven predictors, we further included two treatment indicators (ventilation use, vasopressin use) and five key laboratory variables that differed at baseline but were not selected by LASSO (AST, glucose, sodium, WBC, and total bilirubin). Multicollinearity was assessed using variance inflation factor (VIF) with >5 indicating collinearity. Model discrimination was evaluated by the area under the receiver operating characteristic curve (AUC), and calibration was assessed using a calibration plot. Internal validation was performed to assess model stability.

*Joint subgroup analysis of ALT and RDW:* to further illustrate the combined effect of ALT and RDW on GI hemorrhage, patients were divided into low/high ALT groups and low/high RDW groups by setting the median value of ALT and RDW as threshold, respectively. Then, patients were categorized into high-ALT + low-RDW, low-ALT + low-RDW, high-ALT + high-RDW, and low-ALT + high-RDW groups. Two logistic regression models were employed to determine the exploratory value of ALT combined with RDW in relation to GI hemorrhage.

## 3 Results

### 3.1 GI hemorrhage showed the strongest association with readmission

The study enrolled 2,004 AP patients with 1,559 samples in the non-readmission group and 445 in the readmission group. To identify the complication most strongly associated with readmission, we first looked at the distribution of different complications in non-readmission and readmission groups. The proportions of AKI in the non-readmission and readmission groups were 17.383 and 13.708%, respectively (*P* > 0.05). Two groups had similar proportions of shock (10.648 vs. 10.562%; *P* > 0.05). A difference in the GI hemorrhage was observed in the non-readmission and readmission groups with 24.182 and 48.090% cases, respectively (*P* < 0.05; Table 1). The Phi coefficients for AKI, shock, and GI hemorrhage were −0.041, −0.001, and 0.218, respectively (Table 1). Consistent with the group comparisons, GI hemorrhage showed the strongest positive correlation with readmission (Φ = 0.218, *P* < 0.001), whereas AKI and shock exhibited negligible correlations (both *P* > 0.05). These results suggest that GI hemorrhage is the complication most strongly associated with readmission.

**Table 1:** Distribution of major complications according to readmission status in patients with acute pancreatitis.

Complications	Total (*n* = 2,004)	Non-readmission group (*n* = 1,559)	Readmission group (*n* = 445)	*P*	Phi coefficient
Acute kidney injury				0.066	−0.041
No	1,672 (83.433)	1,288 (82.617)	384 (86.292)		
Yes	332 (16.567)	271 (17.383)	61 (13.708)		
Gastrointestinal hemorrhage				< 0.001	0.218
No	1,413 (70.509)	1,182 (75.818)	231 (51.910)		
Yes	591 (29.491)	377 (24.182)	214 (48.090)		
Shock				0.959	−0.001
No	1,791 (89.371)	1,393 (89.352)	398 (89.438)		
Yes	213 (10.629)	166 (10.648)	47 (10.562)		

### 3.2 Baseline characteristics according to GI hemorrhage status

Given that GI hemorrhage showed the strongest association with readmission, we next compared baseline characteristics between AP patients with and without GI hemorrhage. Expectedly, those with GI hemorrhage accounted for 36.210% of readmissions, higher than those without GI hemorrhage (16.348%; *P* < 0.001). AP patients with GI hemorrhage tended to be younger cigarette users (*P* < 0.05). Besides, there were 28.596% cases of diabetes, 47.885% of hypertension, and 19.628% of mild liver disease among those with GI hemorrhage compared to 22.364, 42.038, and 15.004% respectively in those without GI hemorrhage (*P* < 0.05). A decrease in ALT, AST, sodium, total bilirubin, and WBC levels was observed in AP patients with GI hemorrhage (all *P* < 0.05). Whereas, there was an increase in glucose and RDW levels (all *P* < 0.05). Other parameters including BMI, race, gender, alcohol use, COPD, ventilation use, vasopressin use, bicarbonate, BUN, calcium, chloride, creatinine, platelets, and potassium were not different in the two groups (all *P* > 0.05; Table 2).

**Table 2:** Baseline characteristics of patients with acute pancreatitis according to gastrointestinal hemorrhage status.

Variables	Category	Without GI hemorrhage	With GI hemorrhage	*P*
Age		55.000 [43.000, 69.000]	52.000 [42.000, 63.000]	0.007
BMI		27.635 [24.168, 31.710]	27.336 [23.629, 32.516]	0.492
Race	Non-White	424 (30.007)	197 (33.333)	0.142
	White	989 (69.993)	394 (66.667)	
Gender	Female	711 (50.318)	282 (47.716)	0.288
	Male	702 (49.682)	309 (52.284)	
Alcohol use	Yes	261 (18.471)	125 (21.151)	0.165
Cigarette use	Yes	24 (1.699)	79 (13.367)	< 0.001
Diabetes	Yes	316 (22.364)	169 (28.596)	0.003
Hypertension	Yes	594 (42.038)	283 (47.885)	0.016
COPD	Yes	219 (15.499)	102 (17.259)	0.327
Mild liver disease	Yes	212 (15.004)	116 (19.628)	0.011
Ventilation use	Yes	262 (18.542)	92 (15.567)	0.111
Vasopressin use	Yes	35 (2.477)	14 (2.369)	0.886
ALT, U/L		57.000 [23.000, 192.000]	41.000 [20.000, 96.000]	< 0.001
AST, U/L		57.000 [25.000, 148.000]	45.000 [24.000, 117.000]	0.014
Bicarbonate, mEq/L		25.000 [23.000, 27.000]	25.000 [22.000, 27.000]	0.194
BUN, mg/dL		13.000 [9.000, 20.000]	13.000 [8.000, 20.000]	0.702
Calcium, mg/dL		8.600 [8.100, 9.000]	8.600 [8.100, 9.000]	0.936
Chloride, mEq/L		104.000 [102.000, 107.000]	104.000 [101.000, 107.000]	0.603
Creatinine, mg/dL		0.800 [0.700, 1.100]	0.800 [0.600, 1.100]	0.462
Glucose, mg/dL		104.000 [88.000, 129.000]	110.000 [90.000, 143.000]	0.004
Platelets, K/uL		222.000 [171.000, 287.000]	225.000 [160.000, 298.000]	0.727
Potassium, mEq/L		3.900 [3.600, 4.200]	3.900 [3.600, 4.200]	0.948
RDW, %		13.700 [13.100, 14.700]	14.000 [13.200, 15.100]	< 0.001
Sodium, mEq/L		139.000 [137.000, 141.000]	139.000 [136.000, 141.000]	0.034
Total B, mg/dL		0.800 [0.500, 1.900]	0.700 [0.400, 1.500]	< 0.001
WBC, K/uL		9.000 [6.500, 12.800]	8.300 [6.000, 11.700]	0.004
Readmission	No	1182 (83.652)	377 (63.790)	< 0.001
	Yes	231 (16.348)	214 (36.210)	

GI hemorrhage, gastrointestinal hemorrhage; BMI, body mass index; COPD, chronic pulmonary disease; ALT, alanine transaminase; AST, aspartate aminotransferase; BUN, blood urea nitrogen; RDW, red blood cell distribution width; Total B, total bilirubin; WBC, white blood cell.

### 3.3 Feature screening for GI hemorrhage-related variables

**Figure 1:**
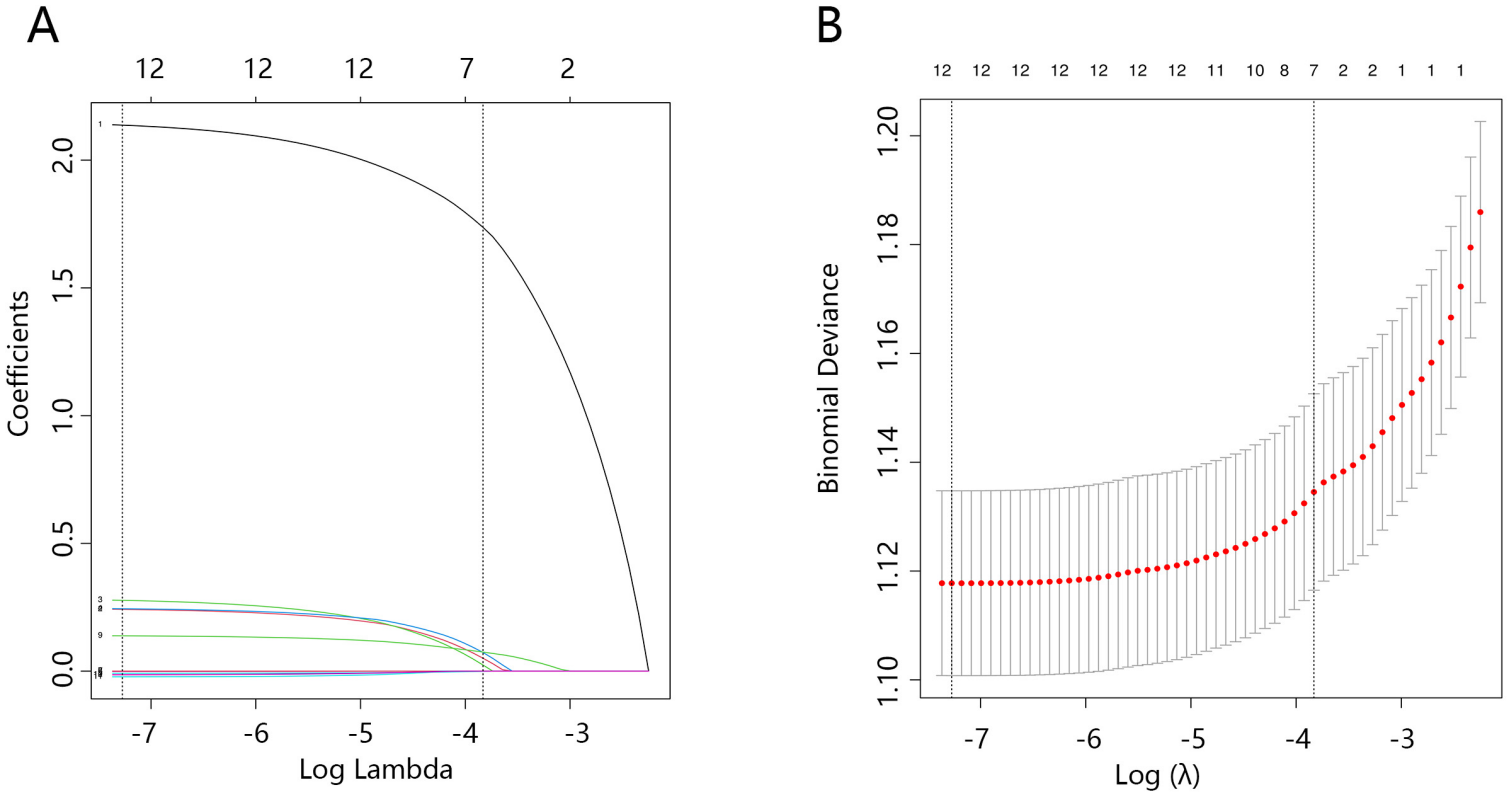
Feature screening by LASSO logistic regression. **(A)** Coefficient profiles of candidate variables. **(B)** Ten-fold cross-validation for selection of the optimal lambda value.

**Figure 2:**
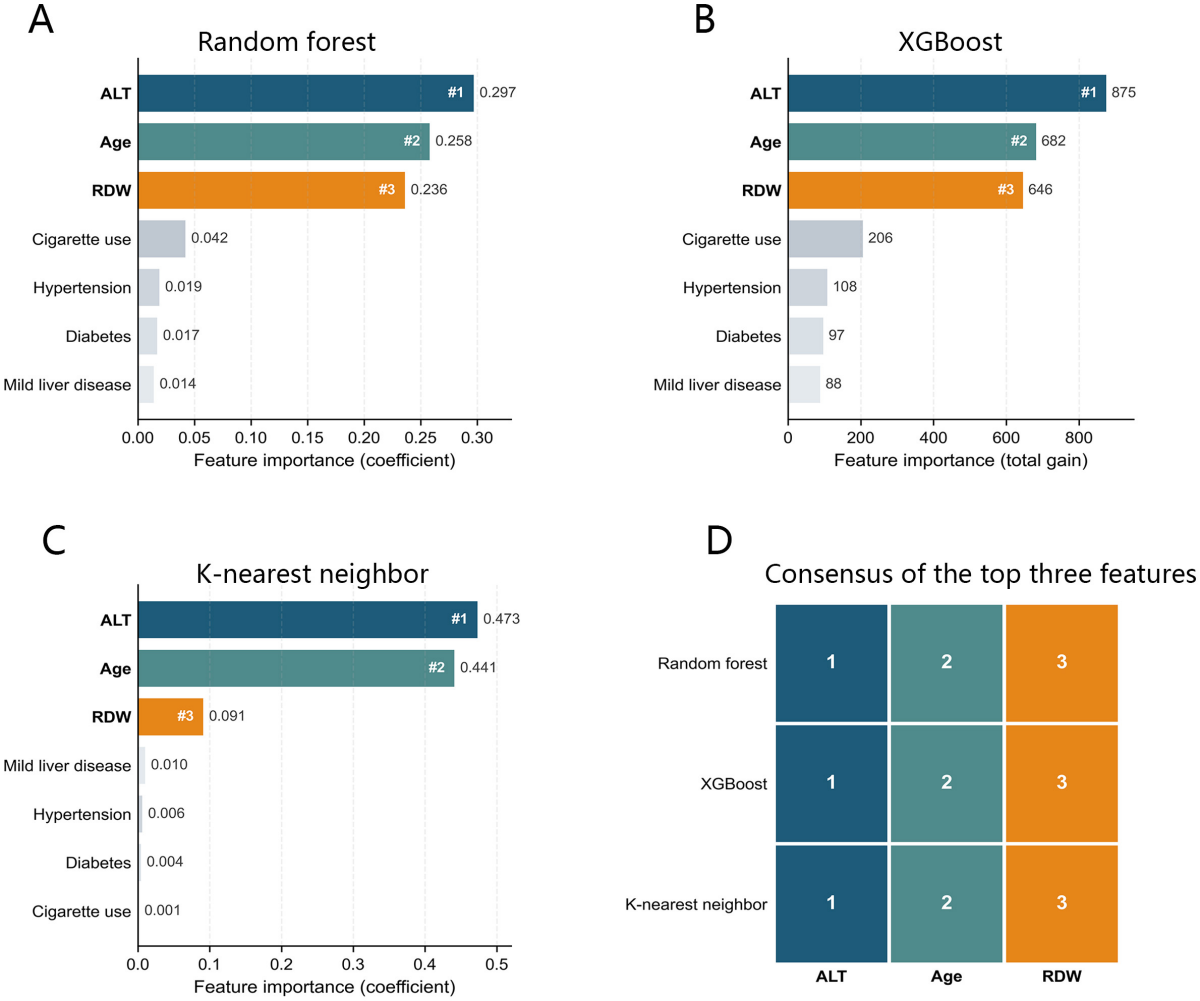
Feature importance ranking across exploratory machine-learning algorithms. **(A)** Random forest. **(B)** XGBoost. **(C)** K-nearest neighbor. **(D)** Consensus of the top three features. ALT, alanine transaminase; RDW, red blood cell distribution width.

Based on the baseline comparison, 12 variables differed significantly between the two groups (*P* < 0.05): cigarette use, diabetes, hypertension, mild liver disease, age, ALT, AST, sodium, total bilirubin, WBC, glucose, and RDW. These 12 variables were then entered into a LASSO logistic regression with 10-fold cross-validation to select the most relevant factors of GI hemorrhage. Seven variables with non-zero coefficients were retained: cigarette use, diabetes, hypertension, mild liver disease, age, ALT, and RDW (AST, sodium, total bilirubin, WBC, and glucose were shrunk to zero; Figures 1A, B). In an exploratory analysis using XGBoost, random forest, and KNN, the top three variables consistently ranked were ALT, age, and RDW (Figures 2A–D).

### 3.4 Associations of ALT and RDW with GI hemorrhage

**Figure 3:**
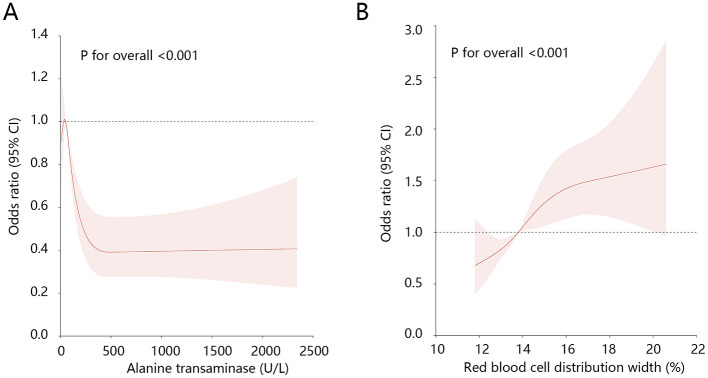
Restricted cubic spline analyses of continuous ALT and RDW in relation to GI hemorrhage. **(A)** ALT. **(B)** RDW. The solid line represents the odds ratio with 95% confidence intervals (shaded areas). The reference value (OR = 1) is set at the median value of ALT and RDW, respectively. Knots were placed at the 10th, 50th, and 90th percentiles. ALT, alanine transaminase; RDW, red blood cell distribution width; 95% CI, 95% confidence interval.

Although age was also among the top important features, it is non-modifiable; we therefore focused on the associations of ALT and RDW with GI hemorrhage. First, RCS regression showed that as the ALT level elevated, the trend of GI hemorrhage decreased (Figure 3A). Inversely, GI hemorrhage risk exhibited an upward trend with the increasing RDW level (Figure 3B).

**Figure 4:**
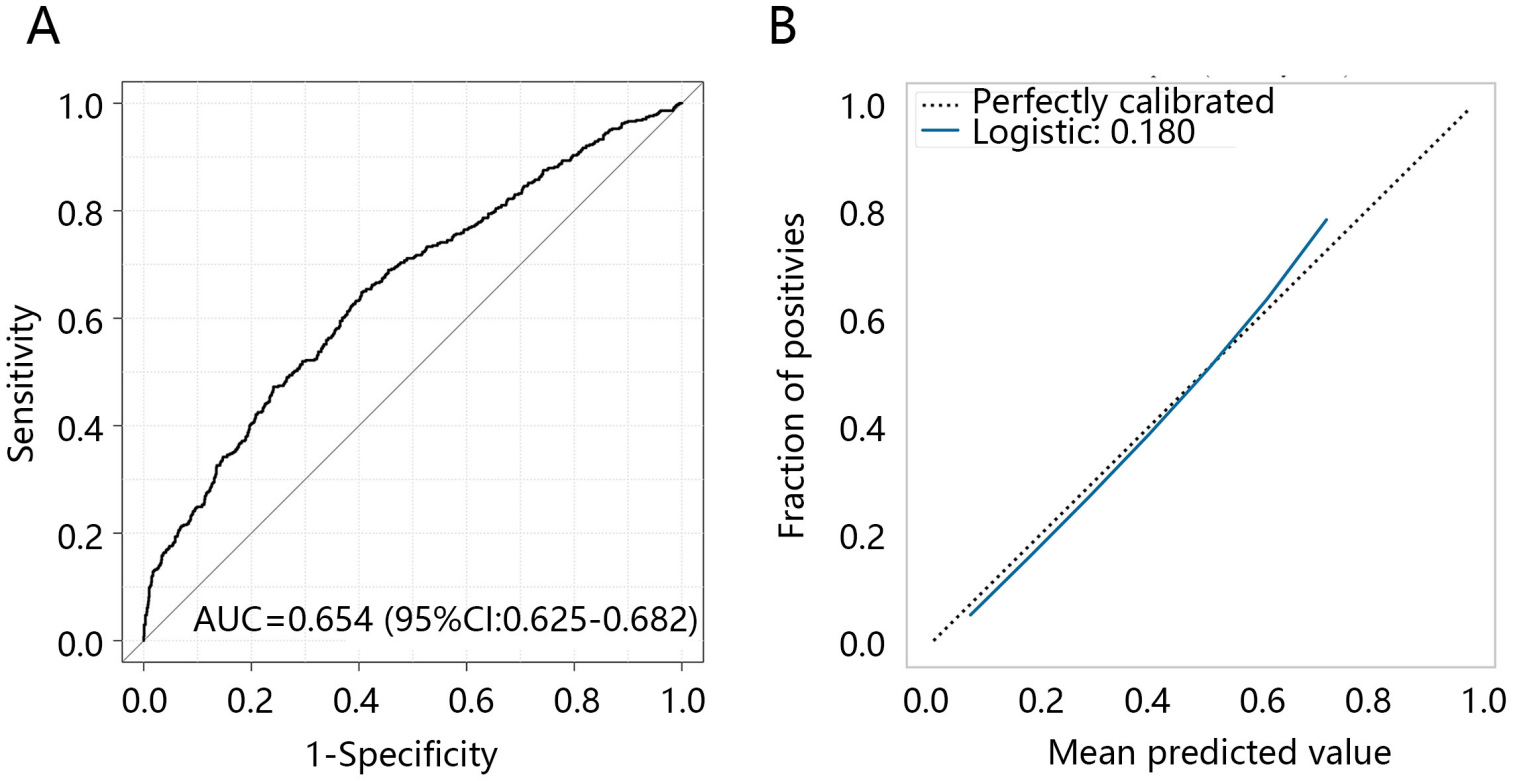
Performance evaluation of the multivariable model. **(A)** Receiver operating characteristic curve. **(B)** Calibration curve. AUC, area under the curve; 95% CI, 95% confidence interval.

In the multivariable analysis using the seven LASSO-selected predictors, RDW elevation was related to an increased risk of GI hemorrhage with an odds ratio (OR) of 1.138 [95% confidence interval (95% CI) = 1.066–1.215; *P* < 0.001]. There was a negative relationship between ALT and GI hemorrhage with an OR of 0.998 (0.997–0.999; *P* < 0.001; Table 3). The model showed acceptable discrimination (AUC = 0.654, 95% CI: 0.625–0.682; Figure 4A) and had good calibration accuracy with a Brier score of 0.180 (Figure 4B).

**Table 3:** Multivariable logistic regression for gastrointestinal hemorrhage.

Predictors	Odds ratio	95% CI-lower	95% CI-higher	*P*
RDW	1.138	1.066	1.215	< 0.001
ALT	0.998	0.997	0.999	< 0.001
Age	0.991	0.984	0.998	0.010
Cigarette use	8.125	4.842	14.246	< 0.001
Diabetes	1.259	0.977	1.618	0.073
Hypertension	1.282	1.02	1.613	0.033
Mild liver disease	1.329	0.998	1.761	0.050

ALT, alanine transaminase; CI, confidence interval; RDW, red blood cell distribution width.

To further evaluate the performance and stability of this multivariable model, we conducted internal model evaluation. Samples were divided into a training set (80%) and a test set (20%). The training set was then used for 5-fold cross-validation, yielding an AUC of 0.622 (0.587–0.657) on the validation sets. The final model achieved an AUC of 0.695 and an accuracy of 0.601 on the test set (Table 4).

**Table 4:** Internal validation and performance of the multivariable model.

Cohort	AUC (95%CI)	Cutoff (95%CI)	Accuracy (95%CI)	Sensitivity (95%CI)	Specificity (95%CI)	F1 score (95%CI)
Training set	0.638 (0.629–0.647)	0.271 (0.258–0.284)	0.618 (0.589–0.646)	0.585 (0.522–0.648)	0.631 (0.567–0.695)	0.463 (0.453–0.474)
Validation set	0.622 (0.587–0.657)	0.271 (0.258–0.284)	0.601 (0.575–0.628)	0.563 (0.478–0.648)	0.617 (0.549–0.685)	0.442 (0.414–0.470)
Test set	0.695 (0.633–0.757)	0.252	0.601 (0.553–0.651)	0.683 (0.594–0.772)	0.568 (0.510–0.626)	0.491 (0.438–0.547)

AUC, area under the curve; CI, confidence interval.

**Figure 5:**
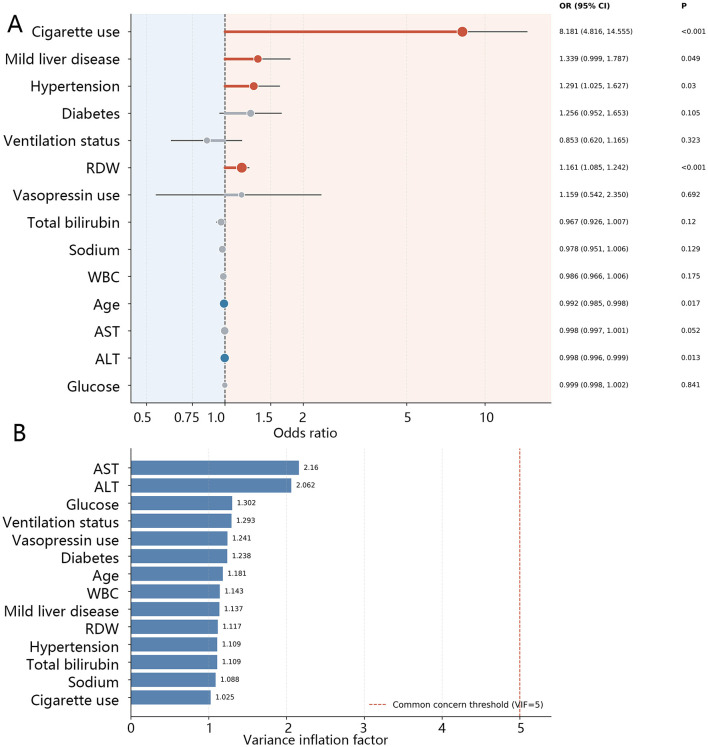
Combined presentation of sensitivity and collinearity analyses. **(A)** Sensitivity analysis shown as an effect-size lollipop plot after additional adjustment for baseline laboratory and treatment variables. **(B)** Collinearity analysis based on variance inflation factors. The dashed red line indicates the common concern threshold of VIF = 5. ALT, alanine transaminase; AST, aspartate aminotransferase; OR (95% CI), odds ratio (95% confidence interval); RDW, red blood cell distribution width; WBC, white blood cell; VIF, variance inflation factor.

In a sensitivity analysis that additionally adjusted for AST, glucose, sodium, total bilirubin, WBC, ventilation status and vasopressin use, the associations remained significant and similar in magnitude (ALT: OR = 0.998, 95% CI: 0.996–0.999; RDW: OR = 1.161, 95% CI: 1.085–1.242; Figure 5A). All the variables in this multivariable regression model had VIF values below 5 (Figure 5B), indicating no multicollinearity, including between ALT and AST.

### 3.5 Joint effect of ALT and RDW on GI hemorrhage

Given the independent associations of ALT and RDW, we next evaluated their combined grouping pattern. The median values of ALT and RDW in the overall cohort were 51.00 U/L and 13.80 %, respectively. Patients were stratified into four groups according to their medians. The proportions of GI hemorrhage in the high-ALT + low-RDW, low-ALT + low-RDW, high-ALT + high-RDW, and low-ALT + high-RDW groups were 19.683, 28.571, 28.829, and 36.650%, respectively (Table S1). Further, we took the high-ALT + low-RDW group as a reference level in the logistic regression models to determine the exploratory role of ALT combined with RDW in relation to GI hemorrhage. In the crude model, compared with the high-ALT + low RDW group, the low-ALT + low-RDW, high-ALT + high-RDW, and low-ALT + high-RDW groups were more vulnerable to developing GI hemorrhage with OR of 1.632, 1.653, and 2.361, respectively (all *P* < 0.05). When the covariates were adjusted, a similar trend was observed with an obvious increased risk of GI hemorrhage in the low-ALT + high-RDW group (OR = 2.315, *P* < 0.001; Table 5).

**Table 5:** Logistic regression models for combined ALT/RDW groups and gastrointestinal hemorrhage.

Predictor	Crude model		Adjusted model	
Predictor	Odds ratio (95% CI)	*P*	Odds ratio (95% CI)	*P*
H-ALT + L-RDW	Reference		Reference	
L-ALT + L-RDW	1.632 (1.205, 2.221)	0.002	1.596 (1.164, 2.197)	0.004
H-ALT + H-RDW	1.653 (1.212, 2.262)	0.002	1.580 (1.144, 2.19)	0.006
L-ALT + H-RDW	2.361 (1.738, 3.222)	< 0.001	2.315 (1.686, 3.194)	< 0.001

H, high; L, low; ALT, alanine transaminase; RDW, red blood cell distribution width.

Crude model: no adjustment; adjusted model: adjusted for age, cigarette use, diabetes, hypertension, and mild liver disease.

## 4 Discussion

This study aimed to identify clinically accessible factors associated with GI hemorrhage to facilitate risk stratification in AP patients. First, we found that GI hemorrhage was the complication most strongly associated with readmission compared with AKI and shock. Through feature selection, ALT and RDW were consistently ranked as the top predictors of GI hemorrhage. The associations remained stable in sensitivity analyses without evidence of problematic collinearity. Finally, the low ALT + high RDW group was associated with a significantly higher risk of GI hemorrhage. However, the predictive model achieved only modest discrimination (AUC = 0.654: 0.625–0.682), indicating limited ability to accurately identify individual patients. This performance precludes any clinical application for risk prediction at this stage.

Readmission after hospitalization for AP occurs in approximately one in five cases (22.2% in our cohort). While previous studies have constructed predictive models for AKI (15, 16) and sepsis (17), we observed that GI hemorrhage was more strongly associated with readmission than either AKI or shock. Hemorrhage, as a type of organ failure, may complicate the course of AP and increase mortality (18). It can be attributed to upper GI ulcers or pseudoaneurysm rupture into the duodenum or the pancreatic duct (19). Non-steroidal anti-inflammatory drugs, ventilation, hypoxia, and stress of severe illness may promote the development of ulcers (20). Therefore, focusing on GI hemorrhage risk factors is clinically relevant for readmission prevention.

The inverse relationship between ALT and GI hemorrhage (OR = 0.998 per unit increase) is intriguing and requires cautious interpretation. ALT is traditionally recognized as a marker of hepatocyte injury; however, low ALT levels have been associated with frailty, sarcopenia, and poor nutritional status in older or critically ill individuals (21, 22). AP patients with lower ALT may have reduced metabolic reserve or subclinical malnutrition, potentially predisposing to stress ulceration and GI bleeding. Conversely, mildly elevated ALT might reflect a more robust acute phase response or better hepatic synthetic function, potentially offering some protection against mucosal injury. However, these hypotheses are speculative, and causality cannot be inferred from observational data.

As a marker of anisocytosis, RDW is related to chronic hepatitis, inflammatory bowel disease, and AP (23–25). Elevated RDW reflects ineffective erythropoiesis, which is often driven by pro inflammatory cytokines such as IL 6 and TNF α, inhibiting erythropoietin production and interfering with iron utilization (26). In AP, systemic inflammation can lead to vascular endothelial injury, coagulation abnormalities, and impaired gastric mucosal blood flow, all of which may predispose to GI hemorrhage (27). Moreover, RDW has been linked to poorer nutritional status and worse clinical outcomes in critical illness (28). Recent studies have also evaluated RDW based composite indices, such as the hemoglobin to RDW ratio and the RDW to albumin ratio, as inflammation related biomarkers in cardiovascular settings (29, 30), supporting the broader relevance of RDW related markers. Thus, the positive association between RDW and GI hemorrhage is biologically plausible, but further mechanistic research is needed.

Joint subgroup analysis (using high ALT + low RDW as the reference group) revealed that the low ALT + high RDW group had the highest odds, whereas the high ALT + high RDW group showed intermediate risks. This pattern suggests that ALT and RDW may capture partially overlapping but distinct pathophysiological pathways—metabolic/nutritional status (ALT) and inflammatory/oxidative stress (RDW). Given the small effect size and the exploratory median-based grouping, the combined assessment of ALT and RDW should be interpreted with caution. The observed risk gradient is hypothesis-generating only and should not be used as a clinical risk stratification tool. Any potential utility requires external validation in independent cohorts before further consideration.

The main strengths are the satisfactory sample size (*n* = 2,004), the systematic feature selection (LASSO and exploratory machine learning ranking), and rigorous validation (discrimination, calibration, and bootstrap internal validation). Sensitivity analysis supports the robustness of the ALT and RDW associations. However, this was a retrospective, single-center study, limiting causal inference and external generalizability. Misclassification of complications or readmission is possible due to reliance on structured database records. The temporal relationship between laboratory testing and GI hemorrhage onset may not be fully captured. Some potentially relevant factors such as AP etiologies and C-reactive protein were not included since excessive missing values. Finally, the median-based grouping and machine-learning ranking were exploratory and require external validation.

In conclusion, among the three complications examined, GI hemorrhage showed the strongest association with readmission in AP patients. ALT and RDW were independently associated with GI hemorrhage, and their combined (low ALT + high RDW) pattern may identify patients at higher risk. However, due to the model's modest performance and the exploratory nature of the findings, prospective and multi-center validation is required before clinical application.

## Data Availability

The raw data supporting the conclusions of this article will be made available by the authors, without undue reservation.
