# Supplementary material for: Association of red blood cell distribution width combined with alanine aminotransferase with gastrointestinal hemorrhage in patients with acute pancreatitis: a retrospective MIMIC-IV study
Source: Front Med (Lausanne). 2026 May 28;13:1777755. doi: 10.3389/fmed.2026.1777755 (PMC13253470; doi:10.3389/fmed.2026.1777755)
Supplement: Supplementary file 1 [file Table_1.docx]

| **Table S1** The distribution of GI hemorrhage in different combined groups. | | | | | |
| --- | --- | --- | --- | --- | --- |
|  | High-ALT+ Low-RDW | Low-ALT+ Low-RDW | High-ALT+ High-RDW | Low-ALT+ High-RDW | P |
| GI hemorrhage |  |  |  |  | <0.001 |
| No | 355 (80.317) | 350 (71.429) | 316 (71.171) | 261 (63.350) |  |
| Yes | 87 (19.683) | 140 (28.571) | 128 (28.829) | 151 (36.650) |  |

Abbreviations: GI hemorrhage, gastrointestinal hemorrhage; ALT, alanine transaminase; RDW, red blood cell distribution width.
